# Binding of Elementary Bodies by the Opportunistic Fungal Pathogen *Candida albicans* or Soluble β-Glucan, Laminarin, Inhibits *Chlamydia trachomatis* Infectivity

**DOI:** 10.3389/fmicb.2018.03270

**Published:** 2019-01-14

**Authors:** Michael D. Kruppa, Jeremy Jacobs, Kelsey King-Hook, Keleigh Galloway, Amy Berry, Jennifer Kintner, Judy D. Whittimore, Rolf Fritz, Robert V. Schoborg, Jennifer V. Hall

**Affiliations:** ^1^Department of Biomedical Sciences, Quillen College of Medicine, East Tennessee State University, Johnson City, TN, United States; ^2^Center for Infectious Disease, Inflammation and Immunity, Quillen College of Medicine, East Tennessee State University, Johnson City, TN, United States

**Keywords:** *Chlamydia trachomatis*, *Candida albicans*, bacterial-fungal interactions, co-culture, normal flora, opportunistic infections

## Abstract

Microbial interactions represent an understudied facet of human health and disease. In this study, the interactions that occur between *Chlamydia trachomatis* and the opportunistic fungal pathogen, *Candida albicans* were investigated. *Candida albicans* is a common component of the oral and vaginal microbiota responsible for thrush and vaginal yeast infections. Normally, *Candida* exist in the body as yeast. However, disruptions to the microbiota create conditions that allow expanded growth of *Candida*, conversion to the hyphal form, and tissue invasion. Previous studies have shown that a myriad of outcomes can occur when *Candida albicans* interacts with pathogenic bacteria. To determine if *C. trachomatis* physically interacts with *C. albicans*, we incubated chlamydial elementary bodies (EB) in medium alone or with *C. albicans* yeast or hyphal forms for 1 h. Following incubation, the samples were formaldehyde-fixed and processed for immunofluorescence assays using anti-chlamydial MOMP or anti- chlamydial LPS antibodies. Replicate samples were replenished with culture medium and incubated at 35°C for 0–120 h prior to fixation for immunofluorescence analysis or collection for EB infectivity assays. Data from this study indicates that both *C. trachomatis* serovar E and *C. muridarum* EB bind to *C. albicans* yeast and hyphal forms. This interaction was not blocked by pre-incubation of EB with the *Candida* cell wall components, mannan or β-glucans, suggesting that EB interact with a *Candida* cell wall protein or other structure. Bound EB remained attached to *C. albicans* for a minimum of 5 days (120 h). Infectivity assays demonstrated that EB bound to *C. albicans* are infectious immediately following binding (0h). However, once bound to *C. albicans*, EB infectivity decreased at a faster rate than EB in medium alone. At 6h post binding, 40% of EB incubated in medium alone remained infectious compared to only 16% of EB bound to *C. albicans*. Likewise, pre-incubation of EB with laminarin, a soluble preparation of β-glucan, alone or in combination with other fungal cell wall components significantly decreases chlamydial infectivity in HeLa cells. These data indicate that interactions between EB and *C. albicans* inhibit chlamydial infectivity, possibly by physically blocking EB interactions with host cell receptors.

## Introduction

Trillions of commensal microbes reside in the human body at all times. While a diverse microbiota has been correlated with health, some studies indicate that microbial interactions can alter the pathogenesis of infection (Baquero and Nombela, 2012; D’Argenio and Salvatore, 2015; Krezalek et al., 2016; Ziklo et al., 2016). For example, respiratory viruses create an environment that is favorable for the growth of bacterial opportunistic pathogens, leading to infections such as otitis media and bacterial pneumonia (McCullers, 2006; Armbruster et al., 2010). Co-infection with sexually transmitted pathogens can increase the transmission of HIV (Rotchford et al., 2000). Thus, interactions between pathogenic and/or commensal microbes represent an understudied facet of human health and disease. In this study, we examined interactions between the bacterial pathogen, *Chlamydia trachomatis* and the opportunistic fungal pathogen, *Candida albicans*.

*Chlamydia trachomatis* is the leading bacterial sexually transmitted infection, causing 1.6 million reported cases in the United States in 2016 (Centers for Disease Control and Prevention, 2017). Genital infections with *Chlamydia* are usually benign with many being asymptomatic or causing uncomplicated cervicitis in women and urethritis in men. However, if untreated these infections can ascend the genital tract leading to pelvic inflammatory disease, ectopic pregnancy and infertility (Peipert, 2003). *Chlamydia* are Gram-negative obligate intracellular bacteria, which exist in the host in two distinct morphological forms. Transmission occurs when the infectious, condensed, extracellular form, the elementary body (EB) enters into the host cell by endocytosis. Once inside the host cell the EB convert to the non-infectious, intracellular, metabolically active, vegetative form called the reticulate body (RB). The RB replicate inside of a modified host vacuole called the inclusion before converting back into EB. Infectious EB are released from the host cell by host cell lysis or extrusion and lysis of the inclusion (Elwell et al., 2016).

*Candida albicans* is a common opportunistic pathogen in the oral and vaginal microbiota and is responsible for vaginal yeast infections and thrush (Mayer et al., 2013). Normally, *C. albicans* exist in the body as a yeast member of the fungal microbiome. However, when given the opportunity, such as in the case of immunosuppression or antibiotic usage, *C. albicans* can convert to an invasive hyphal form that damages the mucosal tissue (Mayer et al., 2013). Invasive *Candida* infections can lead to life threatening fungal sepsis in immunosuppressed individuals. *Candida* have a complex cell wall. The outer layer of the *Candida* cell wall is comprised of mannose polymers, called mannans, that are bound to proteins embedded in the cell wall. The inner layer of the cell wall is primarily composed of β-1,3-glucan and β-1,6-glucan. A small portion of the cell wall is composed of chitin. These structures are critical to the structural integrity of the *C. albicans* cell wall in both the yeast and hyphal forms. Additionally, both mannans and β-glucans are immune stimulatory molecules that are recognized by a variety of host pattern recognition receptors, especially C-type lectin receptors (Gow et al., 2012).

Several previous studies have shown that both symbiotic and antagonistic *Candida*/bacterial interactions occur depending upon the environmental conditions and species involved. *Pseudomonas aeruginosa, Escherichia coli*, and *Acinetobacter baumannii* as well as several other bacteria have been shown to inhibit growth of *Candida* hyphal forms by varying known and unknown mechanisms (Hogan and Kolter, 2002; Peleg et al., 2008, 2010). For example, *Pseudomonas aeruginosa* forms biofilms on *Candida albicans* hyphae, but cannot bind to yeast (Hogan and Kolter, 2002). *P. aeruginosa* releases several soluble compounds including phospholipase C, phenazines and quorum sensing molecules that inhibit the growth of *Candida* hyphal forms, decreasing *Candida*’s virulence (Morales et al., 2013). Conversely, *Candida sp.* inhibit the growth of *Neisseria gonorrheae* via an unknown mechanism (Kaye and Levison, 1977). Fungal/bacterial interactions can also increase microbial pathogenesis. Simultaneous infections of *E. coli* and *C. albicans* in mice increased lethality compared to single infections with either organism (Gale and Sandoval, 1957; Peleg et al., 2010). Interaction of *Streptococcus sp*., including pathogenic Group B *Streptococcus*, with *Candida* promotes enhanced biofilm formation and adhesion to host epithelia (Peleg et al., 2010; Pidwill et al., 2018). Binding of *Staphylococcus* to *Candida* hyphae promotes dissemination of the bacteria into the blood stream of mice (Peters et al., 2012; Kong et al., 2015). Other studies suggest that *Candida* can serve as a protective reservoir for *Helicobacter pylori*, which has been detected in some, albeit not all, instances of atherosclerosis and Alzheimer’s disease (Honjo et al., 2009; Saniee and Siavoshi, 2015). Thus, several interesting outcomes have been documented with *C. albicans/*bacterial interactions. These interactions have the potential to: (1) inhibit growth/virulence of *Candida* or the bacterial pathogens, (2) provide a reservoir for bacterial survival within the body, or (3) promote bacterial dissemination from the site of mucosal infection.

Kelly et al. (2001) demonstrated that concurrent infection with *C. albicans* and *Chlamydia muridarum* did not alter the immune response or vaginal shedding of either organism in mice. However, this study did not investigate direct interaction of the two pathogens *in vitro* or in a simultaneous *in vivo* inoculation (Kelly et al., 2001). Interestingly, dissemination of *Chlamydia* *sp*. from the site of primary infection to distal sites in the body has been reported. Chlamydial DNA has been isolated from the synovial fluid of patients suffering from *Chlamydia*-induced reactive arthritis (Baillet et al., 2015). Dissemination of *Chlamydia pneumoniae* has been associated with atherosclerosis and Alzheimer’s disease (Kuo and Campbell, 2003; Shima et al., 2010). The mechanisms chlamydiae use to disseminate from the site of primary infection throughout the body have not been fully investigated. Given the myriad of medically relevant outcomes that have been reported from investigations of *Candida*/bacterial interactions and the high probability that *Candida* and *Chlamydia* encounter one another in host genital tracts, we sought to determine if direct interactions between *C. trachomatis* and *C. albicans* occur.

## Materials and Methods

### Culture of *Candida*, *Chlamydia*, and Cervical Epithelial Cell Lines

*Candida albicans* SC5314 cultures were routinely cultured overnight at 30°C in YPD (1%yeast extract, 2% peptone, 2% dextrose). Overnight cultures of *C. albicans* were washed with sterile dH_2_O and counted before transferring cells to medium 199 [M199 (9.5 g medium with Earles salts and L-glutamine and without sodium bicarbonate, 18.7 g Tris-HCL in 1L dH_2_O, pH 7.5 or pH 4.5) [Mediatech] at 37°C. *Chlamydia trachomatis* Serovar E and *C. muridarum* Weiss stocks were prepared in Hec-1B cells grown in bead culture as previously described (Guseva et al., 2007). HeLa cells were maintained in Modified Eagle’s Medium (MEM; Gibco) supplemented with 10% FBS, and gentamicin (Gibco). Short tandem repeat profiling was performed by the American Type Culture Collection to authenticate the identity and/or origin of all cell lines used in this study (data not shown). All cell lines were tested for *Mycoplasma* by PCR and found to be free of contamination (data not shown).

### Mannan, β-Glucan, and Laminarin

Fungal cell wall components, mannan, glucan phosphate (β-glucan) or laminarin were prepared in ddH_2_O (1 mg/ml). Mannan was isolated from *Candida albicans* (Kruppa et al., 2011). The β-glucan phosphate was a kind gift from Dr. David Williams at East Tennessee State University. It was prepared from *Saccharomyces cerevisiae* (Williams et al., 1991). Laminarin was purchased from Carbosynth, United States. Purification and activity validation of these biomolecules have been previously described (Williams et al., 1991; Adams et al., 2008; Kruppa et al., 2011; Smith et al., 2018).

### *Candida*/*Chlamydia* Binding Assay

*Candida albicans* SC5314 (1 × 10^5^ cells/sample) was cultured as yeast for 3–6 h in M199 (pH 4.5) medium at 37°C. The yeast were then mixed with 3 × 10^5^ *C. trachomatis* Serovar E or *C. muridarum* EB or 2SPG (0.2 M sucrose, 0.02 M phosphate buffer, and 5 mM l-glutamine) and plated onto coverslips. Alternatively, *C. albicans* was cultured on FBS-coated plastic coverslips for 3–6 h in M199 (pH7.5) medium at 37°C to promote hyphal formation. The *Candida* cultures or plastic coverslips were overlaid with 3x10^5^ *EB* or 2SPG. Following combination of *Candida* yeast or hyphal cultures with EB, the samples were incubated at 35°C for 1 h. The cultures were then washed vigorously three times with PBS to remove unbound EB and fixed with methanol, formaldehyde or glutaraldehyde. In a subset of experiments, EB were overlaid onto plastic coverslips and incubated for 1 h at 35°C, but the inoculum was not removed from the well following incubation. This no-wash (NW) control was replenished with the appropriate medium as described below and harvested for EB titer analysis. Replicate samples were replenished with culture medium (M199 ± 5 μg/ml Amphotericin B or M199 or MEM ± 1 mM glucose-6-phosphate/1% FBS) and incubated for 24–120 h before fixation for IFA or harvest for EB titer analysis. In some experiments, EB were pre-incubated (1 h, 37°C) with fungal cell wall components (1 mg/ml), mannan, glucan phosphate (β-glucan) or laminarin alone or in combination prior to incubation with *Candida* cultures.

### Immunofluorescence Assays and Confocal Microscopy Analysis

Methanol or formaldehyde-fixed samples were stained with BioRad anti-MOMP or LPS Pathfinder stain or with a mouse anti-*Chlamydia* MOMP antibody (Abcam BIOD)/rabbit-anti-mouse Alexa Fluor 488 combination. *C. albicans* was either stained with aniline blue or visualized by differential interference contrast (DIC) microscopy. Samples were examined and imaged using a Zeiss Axiovert microscope and Zen 2012 software. Intensity of fluorescent staining/area (um^2^) was measured by imaging 5 random fields from triplicate experiment samples (*n* = 15) at 200× magnification using set exposure conditions. Confocal images of chlamydial inclusions were captured on a Leica TCS SP8 with Leica LASX software at 1000× magnification. Imaris imaging and Leica LASX software were used to analyze the images.

### Transmission Electron Microscopy

*Candida albicans* or *C. albicans/C. trachomatis* cultures were fixed with glutaraldehyde in 0.1 M Cacodylate buffer (EM Sciences) for 24 h. The monolayers were then collected, agar enrobed, and stained with osmium tetroxide and uranyl acetate before alcohol and propylene oxide dehydration (Wyrick et al., 1994). Dehydrated samples were embedded in Spurr’s low viscosity resin (EM Sciences Kit 14300) to improve penetration of the medium into the *Candida* cell wall (Taff and Andes, 2013). Thin sections were examined on a Tecnai 10 (FEI) transmission electron microscope operating at 60–80 kV.

### Percent Infectivity

Elementary bodies were pre-incubated (1 h, 37°C) in 2SPG alone or containing 1 mg/ml fungal cell wall components, mannan, β-glucan, or laminarin alone or in combination. Following exposure to the cell wall components, HeLa 229 cells were inoculated with the EB/cell wall component mixtures for 1h at 35°C. The inoculum was aspirated from the infected cultures and the cells were replenished with MEM+10% FBS. At 48hpi, the samples were fixed with methanol and stained for chlamydial inclusions using Pathfinder anti-*Chlamydia* MOMP stain (BioRad). Cell nuclei were stained with DAPI (ThermoFisher). Chlamydial inclusions and host cell nuclei were visualized on a Zeiss Axiovert Discovery Microscope at 400× magnification. In each experiment, the number of inclusions and nuclei were counted in 15 random fields from triplicate samples and the average percentage of infected cells was calculated per condition. A high dilution of EB was used in these studies to make counting the percentage of infected cells more accurate.

### EB Titer Analysis

Triplicate *C. albicans*, *C. trachomatis*, *C. albicans/C. trachomatis* cultures, prepared as described in section “*Candida*/*Chlamydia* Binding Assay”, were scraped into the culture medium and frozen at -80°C. EB released from the cells by freeze/thaw and sonication were diluted in culture medium (MEM + 10%FBS, 0.5 μg/ml cycloheximide, gentamicin, and 5 μg/ml amphotericin B) and used to infect HeLa 229 monolayers grown on coverslips by spin infection (1 h, 1100 ×*g*). Triplicate infected monolayers were incubated for 48 h at 35°C before methanol fixation and staining with Pathfinder anti-*Chlamydia* MOMP stain (BioRad). Total inclusions were counted on each coverslip using a Zeiss Axiovert Discovery Microscope at 200x magnification. The average number of inclusion forming units (IFU)/ml was calculated per sample and experimental condition.

### Statistical Analysis

All experiments contained three biological replicates and were independently repeated a minimum of three times. Values from independent experiments were averaged and presented as the mean ± the standard error of the mean (SEM). Means were compared by Analysis of Variance (ANOVA) and independent two-sample *T*-tests using MiniTab Version 17 Statistical Software and Microsoft Excel. Comparisons with *p*-values ≤ 0.05 were considered significantly different.

## Results

### *Chlamydia* Elementary Bodies Bind to the Surface of *Candida albicans* Yeast and Hyphal Forms

**FIGURE 1:**
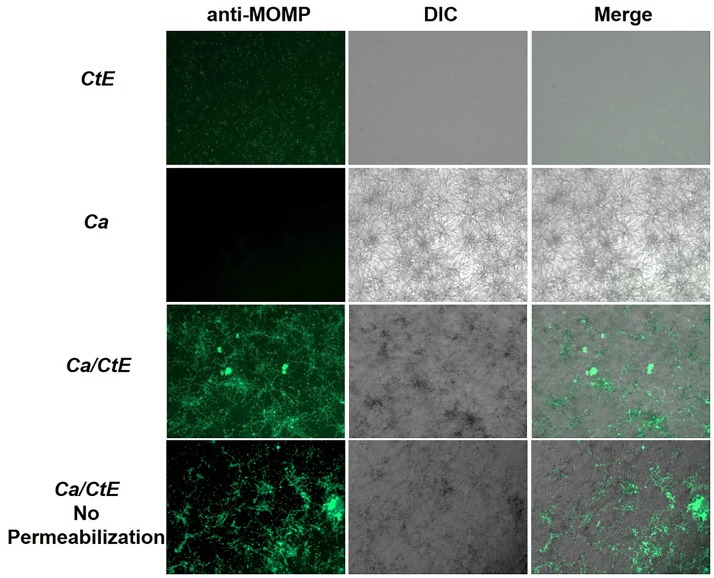
*Chlamydia trachomatis* EB bind to the surface of *Candida albicans*. FBS-coated coverslips were inoculated with *C. albicans* or medium alone and incubated 3 h prior to exposure to EB (Ca/CtE or CtE) or 2SPG *(Ca).* Following incubation of 1 h, the cultures were washed, replenished with medium and incubated for 24 h before collection for IFA by fixation with methanol or formaldehyde (No permeabilization). Samples were stained with Pathfinder anti-*Chlamydia* MOMP stain and visualized 100× magnification.

**FIGURE 2:**
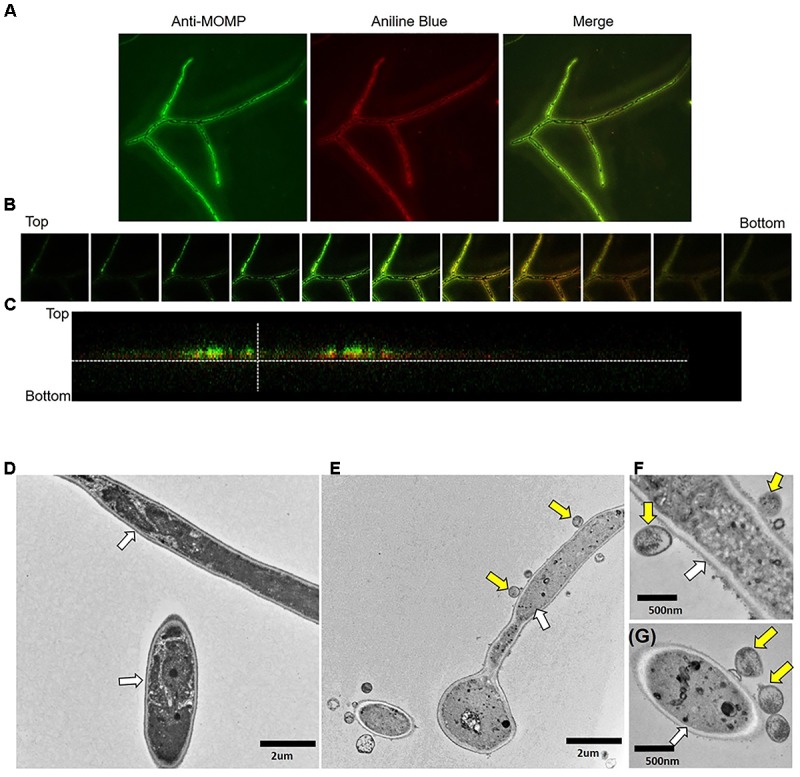
Confocal and transmission electron microscopy imaging of *C. albicans*/*C. trachomatis* cultures. *C. albicans* hyphal cultures were prepared alone or exposed to EB and processed for IFA and TEM visualization as described in the methods. **(A)** Confocal image of Ca/CtE culture at 100x magnification. **(B)** Individual Z sections taken at 30 micron intervals throughout the depth of the sample. Bottom denotes the coverslip. **(C)** Cross-section of the confocal image presented in **(A)**. Green: Pathfinder anti-*Chlamydia* MOMP stain, Red: Aniline blue stain for *Candida.* **(D)** TEM micrograph of *Ca* culture at 7000× magnification. **(E)** TEM micrograph of Ca/CtE culture at 7000× magnification. **(F,G)** TEM micrographs of Ca/CtE cultures at 20000× magnification. Yellow Arrows: *C. trachomatis* EB, White Arrows: *Candida* cell wall.

We sought to determine if *Chlamydia* EB physically interacts with *C. albicans* by examining EB binding to yeast or hyphal forms. We first assessed EB binding to *Candida* hyphal forms. *Candida* yeast or medium alone was seeded onto FBS-coated coverslips and incubated for 3h under conditions that promote hyphal formation. The *Candida* cultures were then overlaid with *C. trachomatis* serovar E EB or 2SPG such that *Chlamydia* (CtE), *Candida* (Ca) and *Candida albicans*/*Chlamydia trachomatis* (Ca/CtE) samples were prepared. Following incubation, the cultures were washed, replenished with medium and incubated for 24h before fixation with methanol or formaldehyde and processed for IFA to visualize any elementary bodies (EB) bound to the coverslips or *Candida*. These experiments demonstrated that EB bind to *Candida* hyphal cultures and remain bound for 24h (Figure 1). No significant difference in chlamydial staining was observed between samples permeabilized with methanol or not permeabilized, indicating that the observed fluorescence was primarily due to binding of EB on the surface of *C. albicans* (Figure 1, bottom row). A similar result was observed with *C. muridarum* EB binding to *C. albicans* (Supplementary Figure 1). In replicate experiments, Ca/CtE samples were harvested for analysis by confocal and transmission electron microscopy as described in the methods. For confocal microscopy we stained *Candida* with the non-specific stain, aniline blue. As shown in Figure 2A, EB staining co-localized with the aniline blue staining of *Candida* hyphae. The chlamydia staining indicates that the EB are bound to the surface of *C. albicans* as there was a clear separation in the *Chlamydia* and *Candida* staining observed in the digital sections and in cross-sections of Ca/CtE samples (Figures 2B,C). TEM images also demonstrate that EB (Figures 2E–G, yellow arrows) bind to the surface of the *Candida* cell wall (Figures 2D–G, white arrows). Data from these imaging analyses support the IFA data, which indicate that *Chlamydia* EB bind to the surface of *C. albicans* hyphae.

As *C. albicans* usually exists *in vivo* in the yeast form, we examined the ability of EB to bind to *C. albicans* yeast. Yeast cultures or medium alone was mixed with EB or 2SPG in suspension for 1h prior to plating the samples onto FBS-coated coverslips. The yeast were then allowed to attach to the coverslips for 1h before the cultures were washed, replenished with medium, and incubated for 0–120 h (1–5 days) before fixation and IFA analysis. In duplicate samples we repeated the *Candida* hyphae/EB binding study described above, harvesting the cultures 0–120 h (1–5 days) for IFA analysis. In both experiments, a replicate set of Ca/CtE cultures were exposed to Amphotericin B to inhibit overgrowth of the *Candida* biofilm. Data from these studies indicate that EB bound to both *C. albicans* yeast and hyphal forms and remained bound for a minimum of 5 days (120 h, Supplementary Figure 2). Continued binding was most evident in samples in which new *Candida* growth was limited by Amphotericin B.

### *Chlamydia* Elementary Bodies Bound to the Surface of *Candida albicans* Lose Infectivity

**FIGURE 3:**
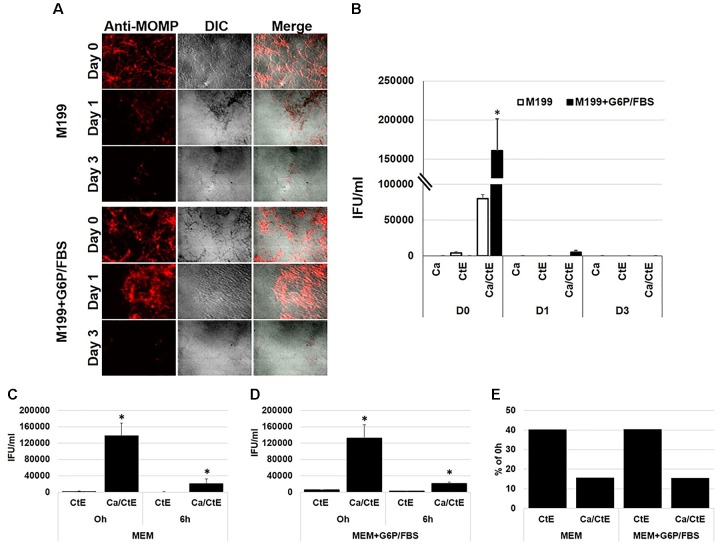
Binding to *Candida albicans* decreases *C. trachomatis* EB infectivity. FBS-coated coverslips were inoculated with *C. albicans* or medium alone and incubated 3h prior to exposure to EB (**Ca/CtE or CtE**) or 2SPG (**Ca**). Following incubation of 1h, the cultures were washed, replenished with MEM or M199 medium ± G6P/FBS. Duplicate sets of samples were fixed and processed for IFA or harvested for EB titer analysis at various times post incubation. **(A)** IFA of Ca/CtE at Day 0, 1, and 3 post incubation in M199 ± G6P/FBS. *Chlamydia* EB -Red: mouse anti-*Chlamydia* MOMP/rabbit-anti-mouse Alexa Fluor 594*, Candida*: DIC 200x magnification. **(B)** EB titers from Ca, CtE and Ca/CtE cultures at Day 0, 1, and 3 post incubation in M199 ± G6P/FBS. EB titers form CtE and Ca/CtE cultures harvested at 0 and 6h post incubation in MEM **(C)** or MEM+G6P/FBS **(D)**. **(E)** Percentage of IFU/ml remaining 6h post incubation compared to 0h for CtE and Ca/CtE cultures in MEM ± G6P/FBS. The data shown represent the means ± SEM of three independent repeats with 3 biological replicates/repeat (*n* = 9). An asterisk (^∗^) indicates a significant difference between CtE and Ca/CtE (*p* ≤ 0.05) as determined by ANOVA and two-sample independent *T*-tests.

We next wanted to determine if EB bound to *Candida* remained infectious. To accomplish this, we set up *Candida* hyphae/EB binding studies as described above in section “*Chlamydia* EB Bind to the Surface of *Candida albicans* Yeast and Hyphal Forms” and the methods. Following the incubation period, the cultures were washed and replenished with either M199 or MEM culture medium. M199 is the standard culture medium used for *C. albicans* culture, whereas MEM is typically used for *C. trachomatis* culture. We chose to replicate the experiments using both types of media so that no experimental artifacts would be observed due to the use of a medium that is not commonly used for either pathogen. Ca, CtE and Ca/CtE cultures were harvested for IFA and EB titer analysis at 0, 1, and 3 days post incubation (Figures 3A,B). As expected, no inclusions were found in *Candida* alone samples. On day 0, there were significantly more infectious EB recovered from the Ca/CtE cultures than the CtE alone. These data again confirm EB binding (Figure 3A) to *Candida* and also indicate that EB remain infectious immediately following binding (Figure 3B). However, by 24h (day 1) post incubation EB bound to *Candida* are no longer infectious (Figure 3B). In fact, EB bound to *Candida* lose a significant amount of infectivity within the first 6h of binding (Figures 3C–E). Remarkably, EB bound to *Candida* lost infectivity at an increased rate compared to EB bound to the coverslip alone. At 6h post binding, 40% of EB incubated in medium alone remained infectious compared to only 16% of EB bound to *C. albicans* (Figure 3E).

Previous studies have shown that maximal viability of *C. trachomatis* EB and RB is dependent on availability of specific energy sources. Omsland et al. (2012) demonstrated that EB require glucose-6-phosphate as an energy source. We wanted to ensure that the observed loss of infectivity by *Candida* bound EB was not simply due to depletion of EB specific energy sources over time. Therefore, we repeated the studies described above using M199 or MEM supplemented with glucose-6-phosphate and 1% FBS (M199 or MEM+G6P/FBS), which has been shown to promote EB viability (Omsland et al., 2012). However, this did not restore or significantly prolong EB infectivity once they were bound to *C. albicans* hyphal cultures (Figure 3). Control samples were included in these experiments, in which the chlamydial EB were not removed and washed away following the hour adsorption period. The controls were replenished with MEM or MEM+G6P/FBS and examined by titer analysis to determine if the EB remained viable in the medium of the course of the experiment. Data from the control samples indicate that 49–79% of EB remained viable in MEM or MEM+G6P/FBS for 6h in the absence of *Candida* (Supplementary Figure 3). These data suggest that specific EB/*Candida* interactions are responsible for the observed decrease in chlamydial infectivity.

### Interaction of *Chlamydia* Elementary Bodies With a Soluble β-Glucan, Laminarin, Inhibits Chlamydial Infectivity in HeLa Cells

**FIGURE 4:**
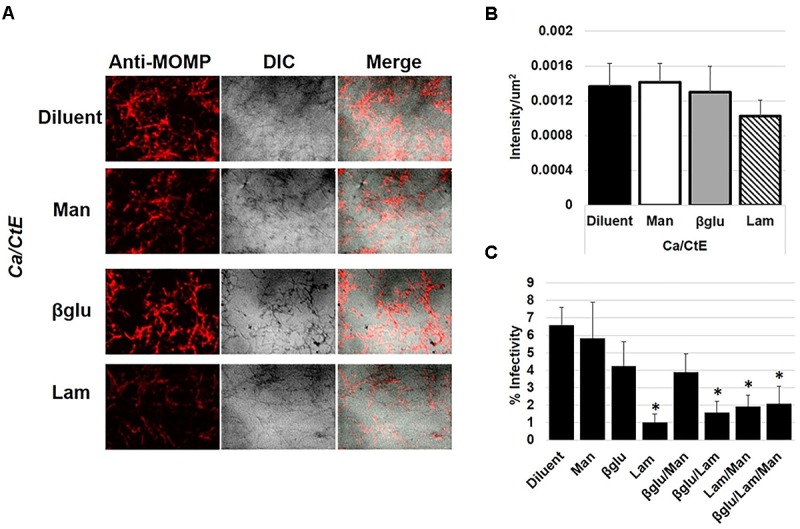
The impact of EB interactions with fungal cell wall components on *C. albicans* binding and infectivity in HeLa cells. *C. trachomatis* EB were incubated with diluent (H_2_O) or fungal cell wall components, mannan (Man), β-glucan (βglu) or laminarin (Lam) for 1h at 37°C prior to incubation with 3h old C. albicans hyphal cultures **(A,B)** or inoculation of HeLa cell monolayers **(C)** as described in the methods. **(A)** Following incubation, Ca/CtE cultures were harvested for IFA. Chlamydia- Red: mouse anti-*Chlamydia* MOMP/rabbit-anti-mouse Alexa Fluor 594, *Candida*: DIC 200× magnification. **(B)** Average intensity/um^2^ of chlamydia staining represented in panel **(A)**. **(C)** The percentage of HeLa cells infected with EB exposed to diluent or fungal cell wall component alone or in combination. The data shown represent the means ± SEM of three independent repeats with 3 biological replicates/repeat (*n* = 9). An asterisk (^∗^) indicates a significant difference between the experimental sample and the diluent control (*p* ≤ 0.05) as determined by ANOVA and two-sample independent *T*-tests.

Interactions of *Chlamydia* with the surface of *Candida* could be mediated via numerous *Candida* cell wall protein or polysaccharide structures. Mannose polymers, mannans, linked to cell wall proteins, as well as 1,3 and 1,6 linked β-glucans comprise a substantial portion of the *Candida* cell wall. Furthermore, these structures are found on both yeast and hyphae (Gow et al., 2012; Lowman et al., 2014). Given that we observed EB binding to both of *Candida*’s morphological forms, these common cell wall structures seemed good candidates for chlamydial binding targets. To examine this possibility, *C. trachomatis* EB were incubated with soluble fungal cell wall components, glucan-phosphate (β-glucan), a commercial preparation of β-glucans called laminarin, or mannan alone or in combination for 1hr (Williams et al., 1991; Adams et al., 2008; Kruppa et al., 2011). Following exposure to the cell wall components, EB were overlaid onto *C. albicans* hyphal cultures prepared as described above. The Ca/CtE cultures were fixed and stained for EB visualization by IFA. Binding of EB to *Candida* was quantified by measuring the fluorescence intensity of chlamydial antibody staining in Ca/CtE cultures prepared with EB exposed to the diluent, mannan, β-glucan or laminarin. Data from these studies indicate that exposure to mannan, β-glucan or laminarin did not significantly block *C. trachomatis* binding to *C. albicans*. However, laminarin exposure modestly decreased chlamydial staining intensity, suggesting it may have a partial effect on binding (Figures 4A,B). In similar experiments, HeLa monolayers were infected with *C. trachomatis* following pre-incubation with diluent, mannan, β-glucan and laminarin alone or in combination. The infected HeLa cultures were harvested for analysis of inclusion development by measuring percent infectivity at 48hpi. Interestingly, pre-incubation of EB with laminarin alone or in combination with β-glucan and mannan significantly decreased chlamydial inclusion formation (Figure 4C, *p* ≤ 0.01) compared to the diluent control. Additionally, we did not observe EB bound to the surface of infected HeLa cells in these experiments, suggesting that laminarin pre-exposure blocked attachment and entry of EB to the epithelial cells (Supplementary Figure 4). These data indicate that interaction of EB with soluble fungal cell wall carbohydrates does not block EB binding to *C. albicans*, but these interactions do inhibit chlamydial infectivity.

## Discussion

Pathogens do not exist in isolation in the genital tract (Lamont et al., 2011). They interact with members of the microbiome as well as neighboring pathogens. There are numerous examples demonstrating that these interactions can influence pathogenesis to benefit or harm the host or the respective microbes (Peleg et al., 2010; Baquero and Nombela, 2012; Mayer et al., 2013; D’Argenio and Salvatore, 2015; Kong et al., 2015 ). Here we present a novel mechanism of chlamydial inhibition by interactions with a common member of the fungal microbiome and opportunistic pathogen, *Candida albicans*. We demonstrated that EB bind *C. albicans* yeast and hyphal morphological forms. Binding to *C. albicans* significantly decreased *C. trachomatis*’ ability to infect human cervical epithelial cells. Moreover, we found that interaction of *C. trachomatis* EB with a soluble preparation of β-glucan, a major component of *C. albicans* cell walls, diminishes chlamydial inclusion development *in vitro*. This is an exciting observation, suggesting that direct binding of chlamydial EB to *Candida* or interaction with shed *Candida* cell wall components may inhibit *C. trachomatis* disease progression.

However, studies have demonstrated that *Chlamydia*-infected women are readily colonized with *Candida* (Jespers et al., 2014; Van Der Pol et al., 2018). Likewise, Kelly, et al. did not observe an effect on vaginal shedding, or immune response to either organism in mice during concurrent infection (Kelly et al., 2001). These studies suggest that simultaneous *Candida* colonization may not negatively impact *Chlamydia* infections *in vivo.* This could be due to differences in the number and/or location of each organism in the genital tract. For example, vaginal *Candida* colonization might have less opportunity to influence an established *Chlamydia* infection in the endometrium. We also observed chlamydial inhibition specifically with laminarin, a soluble preparation of β-glucan. β-glucans are primarily located in the inner layer of the *Candida* cell wall (Gow et al., 2012). Surface exposure of β-glucans does occur, but it is limited to nanoscopic size areas of the cell wall (Graus et al., 2018). Additionally, chlamydial infections *in vivo* occur in the presence of seminal fluid, vaginal secretions and additional members of the vaginal microbiota. Semen is known to enhance transmission of human immunodeficiency virus (Lump et al., 2015; Roan and Münch, 2015). Both semen and the vaginal microbiota have been shown to impact the effectiveness of anti-HIV microbicides (Roan and Münch, 2015; Taneva et al., 2018). Thus, it is possible that: 1) there are not sufficient opportunities for direct inhibitory interaction between EB and *Candida* *in vivo* to significantly decrease *C. trachomatis* infection rates; or 2) chemical and/or physical properties of the *in vivo* environment prevent *C. trachomatis* inhibition by *C. albicans*.

Still, we demonstrated that a commercial preparation of β-glucan was sufficient to inhibit *Chlamydia* *trachomatis* infection in culture. Laminarin is a soluble form of β-glucan isolated from the algae, *Eisenia bicyclis.* It contains short 1,3 linked polysaccharides with minimal 1,6 linked branches. Laminarin has been shown to have a wide range of beneficial properties including tumor inhibition, and the ability to act as an antioxidant or anticoagulant (Miao et al., 1995; Ji et al., 2013; Smith et al., 2018). Most notable though, is its ability to stimulate the immune response. β-glucans activate cytokine production via the C-type lectin receptor, Dectin-1 (Smith et al., 2018). Numerous reports indicate that laminarin has either agonistic or antagonistic effects on Dectin-1 activity. The specific preparation used in this study is a Dectin-1 agonist (Smith et al., 2018).

Pre-exposure of *C. trachomatis* EB to laminarin: (1) modestly decreased *Candida*/*Chlamydia* binding, and (2) inhibited inclusion development in HeLa cells. Several possible mechanisms could be responsible for these observations. First, the physical interactions between EB surface molecules and laminarin may block chlamydial adhesins needed for entry into the host cell. Alternatively, laminarin may bind to host cell surface receptor that is required for chlamydial attachment or entry. It is also possible that because laminarin was not removed from the inoculum, inhibition of *C. trachomatis* may be due to laminarin-induced host cell signaling pathways that negatively impact chlamydial growth. Regardless of the mechanism, laminarin had a strong inhibitory effect on *C. trachomatis*. This interesting observation opens up the possibility that laminarin could be used as a tool for determining the identity and specific functions of unknown chlamydial adhesins that are required for host cell infection. Others have noted that laminarin possesses antimicrobial properties (Pérez et al., 2016). The results presented here suggest that laminarin may function as an anti-chlamydial compound. Furthermore, β-glucan activation of Dectin-1 is a known mechanism of innate immune training (Cheng et al., 2014; Quintin et al., 2014; van der Meer et al., 2015). Studies have shown that innate immune training by β-glucan/Dectin-1 signaling confers non-specific protective effects against subsequent infections (Cheng et al., 2014; Quintin et al., 2014; van der Meer et al., 2015). Thus, theoretically, a β-glucan-based microbicide would have the potential to elicit an immune response in the genital tract that protects the host from infection with *C. trachomatis* or other sexually transmitted pathogens. While these possibilities are very intriguing, we must note a limitation of this study. The inhibitory effects of *Candida/Chlamydia* binding and laminarin pre-exposure were examined using a laboratory strain of *Chlamydia trachomatis* Serovar E. It is possible that clinical isolates will vary in their interactions with *C. albicans* and/or laminarin. Future studies are needed to fully investigate the consequences of *Chlamydia trachomatis* interactions with its fungal neighbor, *Candida albicans*, and the inhibitory actions of laminarin on chlamydial infections.

## Author Contributions

JJ, KK-H, KG, AB, JK, RF, JW, MK, RS, and JH contributed substantially to the design, execution, and/or data collection, and analysis of the experiments within this study. JH drafted the manuscript. All authors contributed to revision and final approval of the manuscript.

## Conflict of Interest Statement

The authors declare that the research was conducted in the absence of any commercial or financial relationships that could be construed as a potential conflict of interest.
