# Supplementary material for: Binding of Elementary Bodies by the Opportunistic Fungal Pathogen Candida albicans or Soluble β-Glucan, Laminarin, Inhibits Chlamydia trachomatis Infectivity
Source: Front Microbiol. 2019 Jan 14;9:3270. doi: 10.3389/fmicb.2018.03270 (PMC6339894; doi:10.3389/fmicb.2018.03270)
Supplement: Supplementary file 1 [file Data_Sheet_1.pdf]

Supplementary Material

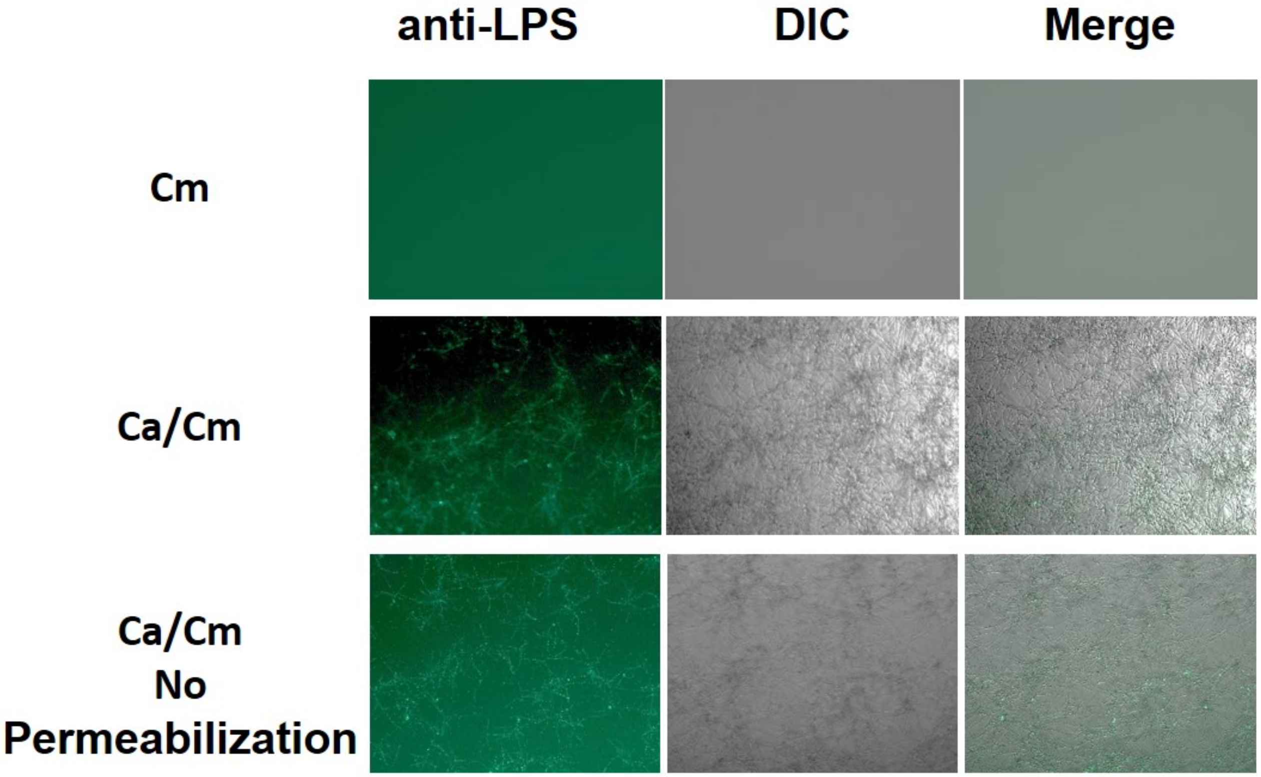

Supplementary Figure 1: *C. muridarum* EB bind to the surface of *Candida albicans*

FBS-coated coverslips were inoculated with *C. albicans* or medium alone and incubated 3h prior to exposure to Chlamydia muridarum EB (Ca/Cm or Cm) or 2SPG *(Ca).* Following incubation of 1h, the cultures were washed, replenished with medium and incubated for 24h before collection for IFA by fixation with methanol or formaldehyde (no permeabilization). Samples were stained with Pathfinder anti-*Chlamydia* LPS stain and visualized 100x magnification.

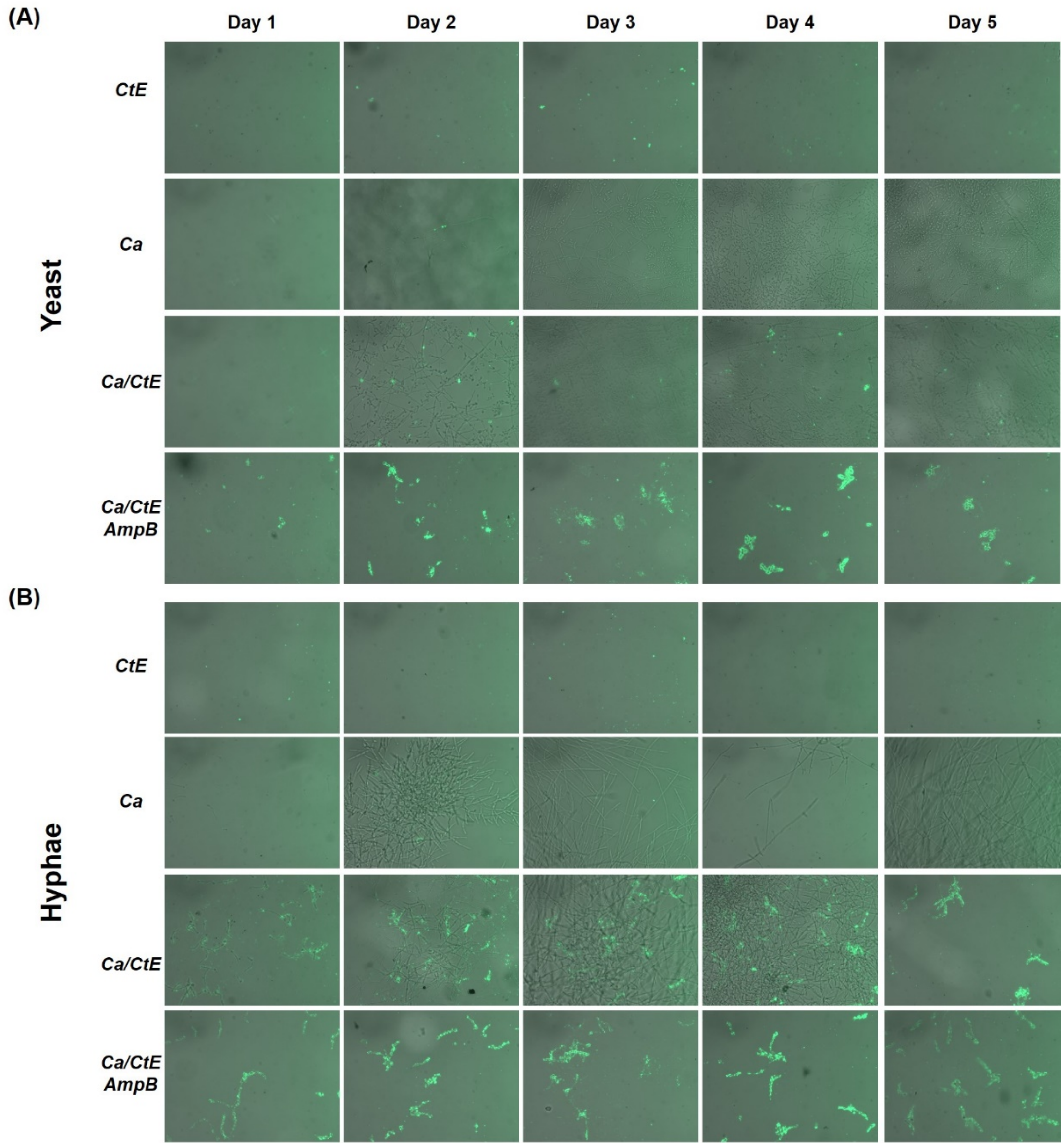

Supplementary Figure 2: ***Chlamydia*****l EB bind to** ***C. albicans*** **yeast and hyphae for extended periods of time.**

A. *C. albicans* yeast cultures (3h) or medium alone was mixed with EB (**Ca/CtE or CtE)** or 2SPG **(Ca)** in suspension for 1h before plating the samples onto FBS-coated coverslips. The yeast were allowed to bind for 1h before the cultures were washed and replenished with medium. **B.** Replicate FBS-coated coverslips were inoculated with Ca or medium alone and incubated 3h prior to exposure to EB (Ca/CtE) or 2SPG (Ca) for 1h. Following the incubation with EB, hyphal cultures were washed and replenished with medium. In both experiments **(A and B)** a replicate set of Ca/CtE cultures were exposed to Amphotericin B to inhibit overgrowth of the *Candida* biofilm. The cultures were harvested for IFA analysis every 24h for 5 days. Samples were stained with Pathfinder anti-*Chlamydia* MOMP stain (Green) and visualized 100x magnification. Candida were visualized by DIC microscopy. Merged images are shown.

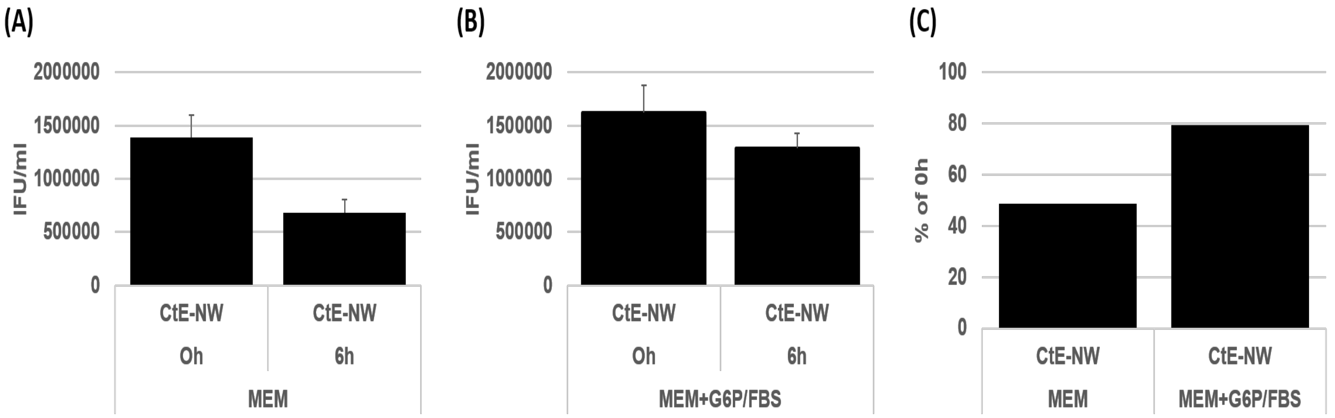

Supplementary Figure 3: Viability of EB in MEM or MEM+G6P/FBS

FBS-coated coverslips were exposed to MEM and incubated 3h prior to exposure to EB. Following a 1h incubation, 500ul of MEM±G6P/FBS was added to each well without removing the inoculum or washing the coverslip **[CtE-No Wash (NW)]**. Triplicate wells were harvested for EB titer analysis at 0 and 6h post EB inoculation in MEM **(A)** or MEM+G6P/FBS **(B). C.** Percentage of IFU/ml remaining 6h post incubation compared to 0h for CtE-NW cultures in MEM±G6P/FBS. The data shown represent the means±SEM of three independent repeats with 3 biological replicates/repeat (n=9). An asterisk (*) indicates a significant difference between CtE-NW in MEM or MEM+G6P/FBS ($p \leq 0.05$) as determined by ANOVA and two-sample independent T-tests.

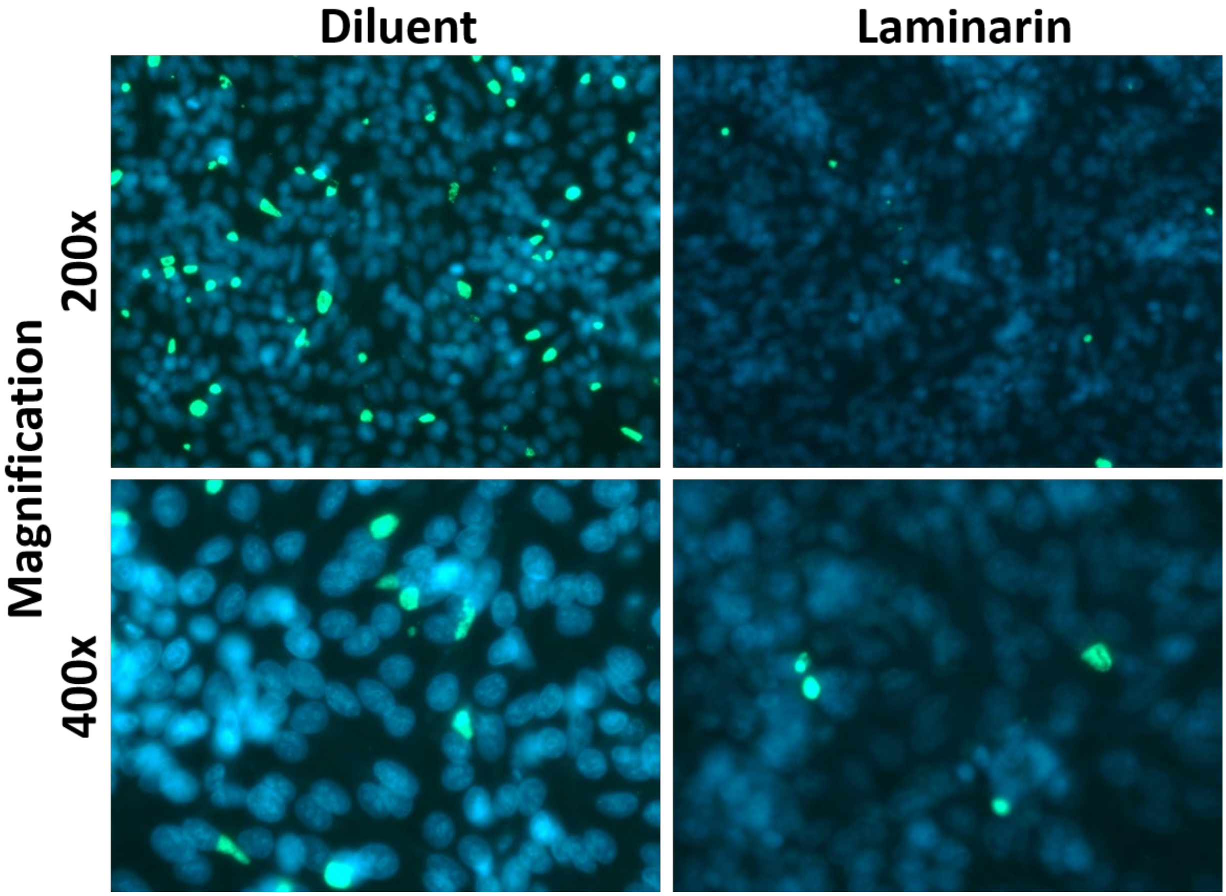

Supplementary Figure 4: The impact of EB interactions with laminarin on *C. trachomatis* infectivity in HeLa cells.

C. trachomatis EB were incubated with diluent (H_2O) or laminarin (Lam) for 1h at 37ºC prior to inoculation of HeLa cell monolayers as described in the methods. At 48hpi, the samples were fixed with methanol and stained for chlamydial inclusions using Pathfinder anti-*Chlamydia* MOMP stain (BioRad). Cell nuclei were stained with DAPI (ThermoFisher). Chlamydial inclusions and host cell nuclei were visualized on a Ziess Axiovert Discovery Microscope at 200x and 400x magnification. Shown are representative images from triplicate samples.
